# Skin Ultrasound Assessment of Patients with Systemic Scleroderma—An Observational Study

**DOI:** 10.3390/jpm14070734

**Published:** 2024-07-08

**Authors:** Camelia Palici, Paulina Lucia Ciurea, Cristina Elena Bita, Andreea Lili Barbulescu, Alesandra Florescu, Anca Emanuela Musetescu, Florentin Vreju, Stefan Cristian Dinescu

**Affiliations:** 1 Doctoral School, University of Medicine and Pharmacy of Craiova, 2 Petru Rares Street, 200349 Craiova, Romania; camicioroianu@yahoo.com; 2 Department of Rheumatology, University of Medicine and Pharmacy of Craiova, 2 Petru Rares Street, 200349 Craiova, Romania; paulina.ciurea@umfcv.ro (P.L.C.); alesandra.florescu@umfcv.ro (A.F.); anca.musetescu@umfcv.ro (A.E.M.); stefan.dinescu@umfcv.ro (S.C.D.); 3 Department of Pharmacology, Faculty of Medicine, University of Medicine and Pharmacy of Craiova, 200349 Craiova, Romania; andreea.barbulescu@umfcv.ro

**Keywords:** systemic scleroderma, ultrasonography, sclerosis, connective tissue diseases, skin diseases

## Abstract

This study aims to analyze the changes in dermal thickness in patients with systemic scleroderma (SSc) in comparison with normal skin and also compare clinical forms with diffuse and limited cutaneous involvement. The study group consisted of female patients diagnosed with SSc with a disease history not exceeding 5 years. The areas of interest for ultrasound examination included the proximal phalanx of the third finger, the second intermetacarpal space, and the extension surface of the lower third of the forearm. The study included 20 patients diagnosed with SSc and 14 controls. SSc patients were subdivided into two subgroups based on the clinical form. Compared to the control group, patients with SSc had higher mean measurements in all three skin areas, with statistically significant differences in the hand and forearm areas. Patients with diffuse SSc displayed, on average, higher skin thickness compared to limited SSc in all skin areas examined, with a statistically significant difference only in the forearm area. Based on disease manifestations, significant differences were observed only with regard to the presence of pulmonary hypertension in the diffuse SSc group. In conclusion, skin ultrasound is a useful and accessible imaging method for diagnosing and quantifying dermal fibrosis in systemic scleroderma.

## 1. Introduction

Systemic scleroderma (SSc) is an autoimmune condition burdened by high rates of morbidity and mortality due to organ involvement that arises during the course of the disease. It appears that the extension of skin sclerosis is a prognostic factor regarding the involvement of vital organs, especially the kidneys and lungs. There is significant scientific evidence suggesting that systemic scleroderma with diffuse cutaneous involvement (dcSSc) presents a major risk for the development of sclerodermic renal crisis and interstitial lung disease. SSc with limited cutaneous involvement (lcSSc) confers an increased risk of developing pulmonary hypertension, but this generally occurs after a more prolonged evolution of the disease compared to dcSSc [1,2]. Steen et al., in a study conducted on 278 patients diagnosed with dcSSc with a disease duration of less than 3 years, demonstrated that improvement in the skin score and a consequent decrease in the rate of skin thickening led to an increase in 5- and 10-year survival rates [3]. Skin ultrasound is a topic increasingly studied in recent years. Considering the relative subjectivity of the modified Rodnan skin score (mRSS), clinicians have sought a more precise and objective method for evaluating the extent of skin changes. By using a high-frequency transducer, it is possible to distinguish the three layers of the skin: epidermis, dermis, and hypodermis. Studies conducted to date have shown that ultrasound can differentiate between the stages of skin involvement in the progression of scleroderma disease [4]. The edematous features of the hand, known as “puffy hands”, are due to edema in the hypodermis, and this suggests that, maybe, in the initial phases of the disease, changes should be sought at this level. Prior research on this topic has reported that greater skin thickness is detected in the early stages of the disease [5]. Also, in the initial stages, US shows the loss of echogenicity due to the edematous component of skin thickening [6,7]. Besides skin thickness and echogenicity, another US feature that can be examined in SSc is skin stiffness, which is measured through elastography techniques such as shear-wave elastography (SWE) [8,9,10,11,12]. 

Multiple studies support the role of high-frequency US for the early detection of skin thickening associated with SSc [13,14,15,16] and the potential to identify pre-clinical dermal involvement [6,16,17]. Studies have shown that US measurements of skin thickness are positively correlated with the mRSS [6,18] and negatively correlated with the duration of the disease [6,18]. A US assessment of skin involvement is sensitive to changes and emerges as a more objective and reliable measurement tool compared to clinical examination through the mRSS [19].

Our study aims to assess the skin thickness of SSc patients through the use of ultrasound in comparison with a control group and to establish whether there are differences with regard to various clinical or laboratory features of the disease.

## 2. Materials and Methods

This observational study was conducted on a study population of 20 patients with SSc, assessed between March and September 2023. This was a single-center study based on data obtained from inpatients selected from consecutive admissions to the rheumatology clinic. All subjects were female with a confirmed diagnosis of systemic scleroderma based on the 2013 ACR/EULAR criteria. In order to better estimate the disease history, the suggestive features considered the best references for disease onset were Raynaud’s phenomenon and skin thickening. Exclusion criteria were patients with a disease history exceeding 5 years and a diagnosis of overlap syndrome. Fourteen healthy subjects were recruited for the control group for comparison. The control group also consisted of only female subjects with a similar age distribution to the SSc group. All participants signed an informed consent form prior to inclusion in the study. Approval for conducting the study was received from the institutional Ethics Committee and registered with nr. 206/20.09.2023.

Regarding the group of individuals with SSc, data related to age, sex, the disease subtype (diffuse cutaneous involvement or limited cutaneous involvement), the type of clinical manifestations present at the time of inclusion in the study, disease duration, and the presence of anti-centromere and anti-SCL70 antibodies were recorded. The presence of skin ulcerations was established based on a physical examination at the time of study inclusion. Pulmonary arterial hypertension was estimated by heart ultrasound measurements. Interstitial lung disease was confirmed based on the results of a lung CT scan.

**Figure 1:**
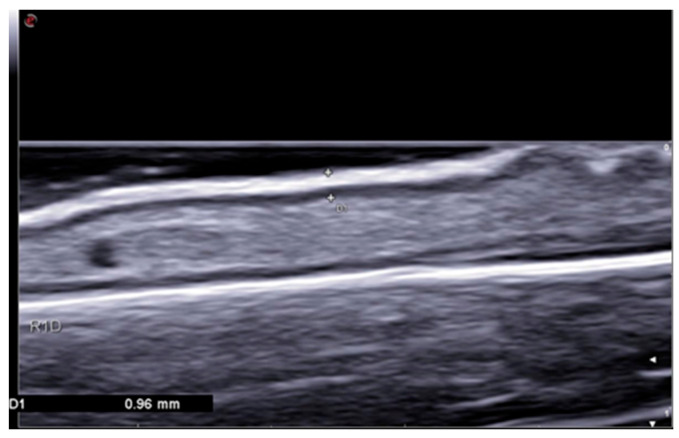
Ultrasonography longitudinal scan at the level of the third phalanx showing a skin thickness measurement that includes epidermal and dermal layers.

Skin ultrasonography was performed by two experienced ultrasonographers (S.C.D. and F.V.) blinded to the study group. Ultrasound evaluation and associated measurements were performed using an Esaote MylabX8 machine with a linear probe frequency of 10–18 MHz. Ultrasound images were obtained as longitudinal sections on the extension surface of the finger, hand, and distal forearm. A thick layer of gel was applied for each scan in order to limit any compression effects. Measurements were taken in three areas of interest in a symmetrical manner (left and right) as follows: Area 1—proximal phalanx of third finger; Area 2—second intermetacarpal space; and Area 3—distal forearm. The examination was performed bilaterally, and the mean value was recorded for each area of interest. The skin thickness was measured by including the epidermal and dermal layers (Figure 1). The values recorded for each region are expressed in millimeters.

A comparison of mean skin thickness measurements was performed using a 2-sample *t*-test or Mann–Whitney U test, depending on the sample data distribution. The analysis included comparisons of skin thickness in the established areas of interest between the control group and SSc group, between the two subtypes of SSc, and with regard to the presence or absence of various disease features. The statistical analysis was performed in R Studio, version 2023.12.1.402.

## 3. Results

The study included 20 patients diagnosed with SSc and 14 controls. The SSc group was subdivided into two subgroups based on the clinical form of SSc as follows: dcSSc—8 cases; lcSSc—12 cases. All subjects included in the study, in both the SSc group and control group, were female. The mean age of patients with SSc was 57.4 years, and that of the control group was 55.86 years. The average disease duration was 1.6 years, with a range of 1 to 5 years. The disease history was recorded as the time since the onset of specific symptoms related to skin changes and/or Raynaud’s phenomenon. It is important to mention that the majority of patients (70%) had a disease history of less than 1 year, and only one patient had a disease history of 5 years.

Table 1 contains an overview of the clinical characteristics observed in the patient cohort with respect to age and the prevalence of various clinical and laboratory features. Pulmonary involvement, esophageal dysmotility, and positive serology were highly prevalent in the SSc group. All patients with SSc displayed Raynaud’s phenomenon, while all patients with dcSSc had a positive serology panel for either anti-Scl70 or anti-centromere antibodies. Overall, 60% of patients were positive for anti-Scl70 antibodies, and 20% of patients were positive for anti-centromere antibodies. All patients were administered calcium channel blockers, while 65% of patients (13/20) were on immunosuppressive therapy, which included mycophenolate mofetil (7 patients), methotrexate (4 patients), or cyclophosphamide (2 patients).

The ultrasound assessment of skin thickness was compared between study groups and the two subtypes of SSc. This analysis was performed for each of the three areas of interest and for the overall mean measurements obtained from all areas examined (Table 2). Compared to the control group, patients with SSc had higher mean measurements in all three skin areas and higher overall means, with statistically significant differences in hand and forearm areas and overall mean measurements. The mean skin thickness among all areas of interest was 1.40 mm in SSc patients and 1.08 in the control group. The highest mean value of skin thickness among SSc patients was recorded in the forearm region, which was also observed in the control group. On comparison between the two subtypes of SSc, there were higher mean thickness measurements for dcSSc in all areas examined and a higher overall mean, with statistically significant differences only in the forearm area. Among all areas of interest between patient subgroups, the highest mean skin thickness measurement was recorded in the forearm area in the dcSSc subtype (Table 2).

**Figure 2:**
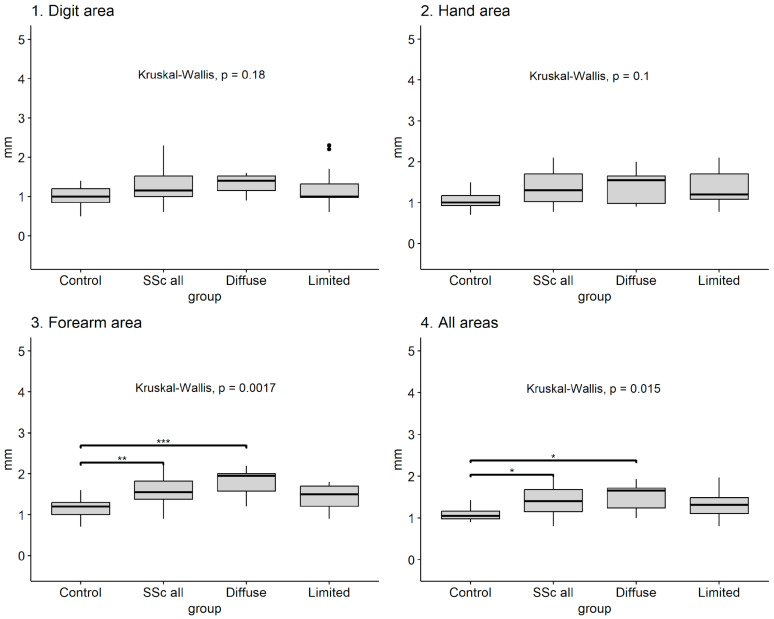
Multiple-group analysis of mean thickness measurements across all areas of interest (* *p* < 0.05, ** *p* < 0.01, *** *p* < 0.001; black dot—outliers).

A multiple-group analysis of mean skin thickness measurements, which also includes a comparison between the control group and subtypes of SSc, is presented in Figure 2. The results of the Kruskal–Wallis test reveal significant differences between all subgroups only in the measurements of the forearm area (*p* = 0.0017) and the overall mean of skin thickness (*p* = 0.015). This was followed by a pairwise comparison analysis, which was performed to identify which subgroup comparison difference had statistical significance (Bonferroni adjustment of *p* value for multiple comparisons). The pairwise comparison revealed differences in the forearm area and overall mean thickness between the control group and both the SSc-all group and the dcSSc group (Figure 2).

Skin thickness measurements were also analyzed with regard to the presence or absence of various clinical and laboratory features (Table 3). For the main SSc study group, there were consistently higher values across all areas for the presence of pulmonary arterial hypertension and esophageal involvement. Importantly, none of these differences had statistical significance. For the dcSSc group, there were consistently higher values across all areas only for the presence of pulmonary arterial hypertension, with statistically significant differences for the digit and hand areas and overall mean skin thickness. No comparison could be performed with regard to the presence of esophageal involvement or positive serology since these features were present in all patients. For the lcSSc group, there were consistently higher values across all areas only for the presence of skin ulcerations, but none of the differences observed had statistical significance.

## 4. Discussion

In rheumatology, ultrasound has proven its utility, especially in detecting structural changes that occur in the joint and periarticular components within inflammatory or degenerative diseases [20]. The applications of this technique even extend to more superficial structures, such as the skin and nails. The use of a transducer with a frequency of 15–20 MHz can depict the trilaminar structure of the skin. In systemic scleroderma, ultrasonography becomes a particularly important technique in assessing changes in the papillary and reticular dermis, even in subclinical stages. Its non-invasive, non-radiating nature and the practical absence of all contraindications allow detailed monitoring of the natural progression of the disease [21].

The extent of fibrotic changes in the skin and the speed at which they occur are negative prognostic factors in systemic scleroderma. The first method of quantifying this progression was the Rodnan score, but this score is susceptible to subjectivity. This aspect has encouraged the use of ultrasonography as an imaging technique to objectively and reproducibly quantify the qualitative and quantitative variations in skin features in systemic scleroderma [22].

The pathophysiology of skin changes in systemic scleroderma is complex. Microcirculation disturbances triggered by the aberrant activation of endothelial cells lead to abnormal responses from other interconnected effector components. Oxidative stress caused by chronic hypoxia generated by repeated vasoconstriction has a stimulating effect on fibroblasts and circulating fibrocytes [23]. The consequence of this is the excessive deposition of fibrillar collagen in the dermis, especially type V collagen alpha 1 and alpha 2, in the early stage of the disease [24]. Type V collagen is stimulated by TGF-beta and other growth factors. It is an important component of the extracellular matrix and contributes to the interconnection of type I collagen fibers. Type V collagen is also found at intercellular junctions and contributes to stabilizing the dermo-epidermal interface [25]. Other locations of this type of collagen with an impact on the pathogenesis of systemic scleroderma include the mesangium and glomerular basement membrane, interalveolar septa, and vascular wall [26].

The focus of research in recent years has been on finding an accessible and cost-effective way to detect early scleroderma changes and applying therapeutic interventions as early as possible in the course of the disease. Subclinical changes in skin involvement can be highlighted by high-frequency ultrasonography. The accurate measurement of skin thickness can differentiate the three evolutionary stages [14,27]. Although not comparable in terms of precision with skin biopsy, skin ultrasound is a helpful tool in identifying structural alterations. It is also a non-invasive, rapid, cost-effective method, much more easily accepted by patients compared to skin tissue sampling [28]. The use of transducers with high frequency (15–18 MHz) and very high frequency (20–30 MHz) allows the detection of changes in echostructure compared to normal skin and can distinguish between the edematous phase and more advanced stages displaying atrophy of the skin [29,30].

Ruaro et al. conducted a study on patients with systemic scleroderma and demonstrated a correlation between circulatory disorders, the level of circulating fibrocytes, and the thickness of the dermis measured by ultrasound at the level of the third finger and the dorsal surface of the hand [13]. This was further confirmed in a study by Sulli et al., who reported that the dermal thickness correlates proportionally with the extent of microcirculation impairment [14], with higher skin thicknesses observed in subjects with a late capillaroscopic pattern and limited cutaneous involvement form. Moore et al. laid the foundation for a score to accurately assess skin fibrosis in systemic scleroderma through an ultrasound evaluation of 17 areas of interest [31]. Although this comprehensive examination method provides more detailed results, it is time-consuming and, for this reason, not efficient for everyday practice. Thus, examining three or five areas of interest, such as the dorsal surface of the finger, hand, and forearm, the chest, the leg, and the extension surface of the foot, is considered sufficient [7].

However, there are debates about the most relevant skin area to examine. Although the skin on the fingers is the first site affected by excessive collagen deposition [32], the relatively small thickness of the dermis and the proximity to the bony surface necessitate the use of very-high-frequency transducers and a highly experienced examiner to make accurate assessments of changes at this level. Routine examination of the dorsal surface of the hand and forearm is preferred, especially in dcSSc, which seems to involve higher dermal thickness than lcSSc [7,27]. Our results also support this observation, since patients with dcSSc showed numerically higher skin thickness than the control group, and this was more evident in the forearm area, for which the difference observed obtained statistical significance (*p* = 0.019).

It is important to define cut-off values for normal subjects in order to establish whether there are meaningful differences while assessing a patient with SSc. Research on this topic, such as the study by Hesselstrand et al., helps to establish some reference points for each area of interest [7]. Sun et al. have determined cut-off values for systemic scleroderma of 1.35 mm for the third finger and 1.26 mm for the forearm [33]. The age of the patient is also an important factor to consider since differences in normal skin thickness can be explained by the physiological phenomenon of the number of collagen fibers reducing with age, a phenomenon more pronounced in women over 50 years old [34]. The lower mean skin thicknesses in the healthy subjects included in our study in comparison to other studies may be explained by the average age of 55.86 years in the control group.

A study on 40 patients with dcSSc demonstrated a statistically significant correlation between cutaneous collagen deposition and the degree of pulmonary fibrosis shown by HRCT [17]. Additionally, these patients had lower DLCO, higher C-reactive protein levels, and a higher EUSTAR-DAI score [18]. In our study, we identified higher values for all three skin areas with regard to the presence of pulmonary hypertension and esophageal involvement, although statistical significance was obtained only for pulmonary involvement in the dcSSc group. Positive serology was also associated with numerically higher values of the overall mean skin thickness, but this was not observed in the subgroup analysis based on clinical form.

Elastography is a complementary examination to cutaneous ultrasound that provides additional information about skin stiffness in scleroderma patients. There are several elastography techniques, with the most commonly used being real-time elastography that displays the results in color codes and shear-wave elastography. This technique is much more precise in assessing skin hardness compared to manual palpation. Real-time ultrasound can also differentiate between the evolving phases of cutaneous fibrosis, distinguishing between the edematous phase and the hardening phase through collagen deposition in the dermis [35].

In the assessment of skin involvement in SSc, US shear-wave elastography measurements demonstrate remarkable sensitivity, specificity, and reliability compared to other semi-quantitative and quantitative methods. Using 6 out of the 17 designated sites for evaluating SSc involvement could be particularly useful for patients with lcSSc, considering that those with dcSSc usually experience an uneven progression of skin thickening [36]. The proximity to the bony interface at the level of the fingers could disrupt the proper functioning of the elastographic algorithm, compromising the accurate assessment of dermal elasticity in this area. This obstacle may hinder a correct representation of digital structures, suggesting that US elastography may not be a reliable tool for evaluating tissue elasticity in sclerodactyly [37].

Studies have provided evidence that measuring skin thickness in systemic scleroderma can be an effective tool in predicting organ involvement, such as pulmonary fibrosis, pulmonary hypertension, and esophageal dysmotility. From this perspective, ultrasound may become an efficient and accessible tool for both diagnostic and prognostic assessments.

In 2022, the World Scleroderma Foundation Working Group developed the first recommendations, which aim to standardize the assessment and reporting of skin US results [38]. In addition to skin thickness, US has other potential applications for the management of SSc patients, including the assessment of joints, lung pathology [39,40,41], the presence and extent of digital ulcerations [42], or associated skin calcinosis [43]. 

Regarding the therapeutic options for skin manifestations in SSc, there is evidence that supports the benefit of several immunosuppressive agents. These include methotrexate, mycophenolate mofetil, rituximab, tocilizumab, cyclophosphamide, or cyclosporine [44,45]. Most clinical trials with a focus on skin involvement include patients with early dcSSc and rely on composite endpoints that include the improvement of the mRSS, such as the American College of Rheumatology Composite Response Index in Systemic Sclerosis (CRISS) [46]. Nevertheless, studies have shown that a change in the mRSS is associated with improvement in organ involvement and can be considered a surrogate response marker [47,48]. In this regard, future research should test the validity of skin US as an objective and sensitive tool for monitoring treatment response after antifibrotic and immunosuppressive agents.

Important limitations of this study arise from the small number of patients with SSc and the absence of male patients, which originates from its very low prevalence in males. Also, most patients included in the study had a disease history of up to two years, which limits comparisons between different disease stages. 

## 5. Conclusions

Patients with systemic scleroderma displayed, on average, greater skin thickness across all areas examined compared to the control group, with higher values in the diffuse cutaneous form. The presence of pulmonary hypertension and esophageal involvement was associated with increased overall skin thickness. Skin ultrasonography emerges as a useful and accessible method for identifying skin changes related to cutaneous fibrosis in systemic scleroderma patients.

## Figures and Tables

**Table 1:** Study population characteristics.

Characteristic	n = 20 ^1^
Age	57.4 (45–79)
Disease history	1.6 (1–5)
Clinical form	
Diffuse	8 (40%)
Limited	12 (60%)
Pulmonary involvement	14 (70%)
Interstitial lung disease	12 (60%)
Pulmonary hypertension	8 (40%)
Esophageal dysmotility	14 (70%)
Skin ulcerations	12 (60%)
Raynaud’s phenomenon	20 (100%)
Serology	16 (80%)
Positive anti-Scl70 antibodies	12 (60%)
Positive anti-centromere antibodies	4 (20%)

^1^ Mean (range); n (%).

**Table 2:** Comparison of skin thickness by US between study subgroups.

	Mean Skin Thickness Measured by US (mm)					
Area of Interest	Control	SSc All	*p* Value	dcSSc	lcSSc	*p* Value
digit ^1^	1.00	1.27	0.09	1.32	1.24	0.648
hand ^2^	1.06	1.37	**0.02**	1.41	1.35	0.740
forearm ^3^	1.17	1.56	**0.001**	1.80	1.40	**0.019**
overall	1.08	1.40	**0.007**	1.51	1.33	0.270

US—ultrasound; SSc—systemic scleroderma; dcSSc—diffuse cutaneous systemic scleroderma; lcSSc—limited cutaneous systemic scleroderma. ^1^ Extension surface of third finger on proximal phalanx; ^2^ extension surface of second intermetacarpal space; ^3^ extension surface of distal forearm; in bold—*p* values < 0.05.

**Table 3:** Comparison of skin thickness by US in relation to clinical and laboratory features.

	Disease Features														
Study Group and Area Examined	Interstitial Lung Disease			Pulmonary Hypertension			Esophageal Involvement			Skin Ulcerations			Positive Serology		
	No	Yes	*p* Value	No	Yes	*p* Value	No	Yes	*p* Value	No	Yes	*p* Value	No	Yes	*p* Value
SSc all															
digit	1.29	1.26	0.9	1.17	1.43	0.2	1.24	1.29	0.9	1.23	1.31	0.7	1.31	1.27	>0.9
hand	1.49	1.30	0.3	1.30	1.48	0.4	1.30	1.41	0.6	1.38	1.37	>0.9	1.50	1.34	0.6
forearm	1.60	1.53	0.7	1.51	1.64	0.5	1.42	1.62	0.3	1.62	1.53	0.6	1.36	1.61	0.2
all areas	1.46	1.36	0.6	1.33	1.52	0.3	1.32	1.44	0.6	1.41	1.40	>0.9	1.39	1.41	>0.9
Diffuse cutaneous SSc															
digit	1.50	1.27	0.2	1.13	1.53	**0.029**	NA	1.33	NA	1.43	1.23	0.3	NA	1.33	NA
hand	1.55	1.37	0.4	1.10	1.73	**0.026**	NA	1.41	NA	1.60	1.23	0.2	NA	1.41	NA
forearm	1.95	1.75	0.3	1.58	2.03	0.066	NA	1.80	NA	1.95	1.65	0.2	NA	1.80	NA
all areas	1.67	1.46	0.3	1.27	1.76	**0.035**	NA	1.51	NA	1.66	1.37	0.2	NA	1.51	NA
Limited cutaneous SSc															
digit	1.22	1.26	>0.9	1.19	1.34	0.7	1.24	1.24	>0.9	1.03	1.35	0.2	1.31	1.21	0.8
hand	1.47	1.23	0.3	1.40	1.24	0.6	1.30	1.40	0.7	1.15	1.45	0.13	1.50	1.27	0.5
forearm	1.49	1.32	0.4	1.48	1.25	0.4	1.42	1.39	0.9	1.28	1.46	0.3	1.36	1.43	0.7
all areas	1.39	1.27	0.6	1.36	1.28	0.8	1.32	1.34	>0.9	1.15	1.42	0.15	1.39	1.30	0.7

SSc—systemic scleroderma; in bold—*p* values < 0.05.

## Data Availability

The dataset is available on request from the authors.
